# Reconstitution of Kidney Side Population Cells after Ischemia-Reperfusion Injury by Self-Proliferation and Bone Marrow-Derived Cell Homing

**DOI:** 10.1155/2013/370961

**Published:** 2013-06-24

**Authors:** Hongbao Liu, Weihui Liu, Shuibing Liu, Qiuhong Meng, Ning Zhang, Hanmin Wang, Rong Li, Limin Wang, Peng Zhang, Shiren Sun

**Affiliations:** ^1^Department of Nephrology, Xijing Hospital, The Fourth Military Medical University, Xi'an 710032, China; ^2^PLA Center of General Surgery, General Hospital of Chengdu Army Region, Chengdu 610083, China; ^3^School of Pharmacy, Fourth Military Medical University, Xi'an 710032, China; ^4^Department of Hepatobiliary Surgery, Xijing Hospital, Fourth Military Medical University, Xi'an 710032, China; ^5^Foreign Language Department, Bethune Military Medical NCO's Academy, Shijiazhuang 050081, China

## Abstract

The aim of this study was to examine the contribution of side population (SP) cells from kidney and bone marrow for reconstitution of kidney SP pools after ischemia-reperfusion injury (IRI). The SP and non-SP cells in kidneys following IRI were isolated and serially assessed by fluorescence-activated cell sorting. The apoptosis, proliferation, phenotype, and paracrine actions of SP cells were evaluated *in vitro* and *in vivo*. Results indicated that the SP cells from ischemic kidney were acutely depleted within one day following renal IRI and were progressively restored to baseline within 7 days after IRI, through both proliferation of remaining kidney SP cells and homing of bone marrow-derived cells to ischemic kidney. Either hypoxia or serum deprivation alone increased apoptosis of SP cells, and a combination of both further aggravated it. Furthermore, hypoxia *in vivo* and *in vitro* induced the increase in the secretion of vascular endothelial growth factor, insulin-like growth factor 1, hepatocyte growth factor, and stromal cell-derived factor-1**α** in kidney SP but not non-SP cells. In summary, these results suggest that following renal IRI, kidney SP cells are acutely depleted and then progressively restored to baseline levels by both self-proliferation and extrarenal source, that is, bone marrow-derived cell homing.

## 1. Introduction

Renal ischemia/reperfusion injury (IRI) is the most common cause for acute kidney injury (AKI) and is associated with high morbidity and mortality [1]. A large number of studies have focused on the endogenous and exogenous mechanisms of kidney repair after ischemic/hypoxic injury [2, 3]. A single step purification method for hematopoietic stem cells, based on the efflux of the DNA binding dye Hoechst 33342, has been reported [4]. The isolated cells were called side population (SP) cells, and they were also found in other nonhematopoietic organs including kidney [5–13]. The kidney SP cells can differentiate into multilineage and ameliorate acute kidney injury [6, 7, 9, 12], suggesting that kidney SP cells are a good target for clinical renal regenerative therapy. Several studies have demonstrated that the kidney SP cells were decreased in the kidney of several kidney injury animal models [7, 9, 13]. The possibility as to whether the depletion of resident SP cells in the injured kidney is provisional or permanent remains to be determined. During renal repair from ischemic injury, the proliferative activity of remaining renal cells increases dramatically and the mobilization of bone marrow-derived cells (BMCs) into the injured kidney improves significantly, which reflects the intrinsic ability of these cells to replace damaged cells with new ones [14–16]. The SP cells, including kidney SP and bone marrow-derived SP, probably have similar responses, as mentioned above, during repair from an ischemic insult, which needs to be determined. Our previous study has reported that, for the animals with renal IRI, the infusion of exogenous kidney SP cells can significantly improve renal function, accelerate mitogenic response, and reduce cell apoptosis [12]. However, the response of endogenous SP cells to renal IRI still remains unknown; especially, the knowledge about proliferation, reconstitution, and paracrine actions of endogenous SP cells after IRI has not been evaluated *in vivo*.

In the present study, therefore, we attempted to investigate serial changes of the SP cell numbers during renal IRI and to validate the relative relevance among proliferation, restoration, and paracrine actions of SP cells.

## 2. Materials and Methods

### 2.1. Animals

C57BL/6 mice (age from 6 to 8 weeks) were provided by the Experimental Animal Center of the Fourth Military Medical University (Xi'an, China). All procedures involving animals were approved by the Institutional Animal Care and Use Committees of the university. Animals bred in the house with constant temperature and humidity, with a 12/12 h light/dark cycle, could freely ingest standard diet and tap water. The results for all experiments had been demonstrated at least six times.

### 2.2. Induction of Renal IRI

Renal IRI was induced in isoflurane anaesthetized female C57BL/6 mice, and rectal temperature was maintained at 37°C. After a mid-abdominal laparotomy, kidneys were exposed and left kidney pedicles were clamped with atraumatic vascular microclamps for 30 min followed by clamp release to allow reperfusion, as described earlier [12, 17]. Before and 1, 3, and 7 days after unilateral renal IRI, kidney SP and non-SP cells were, respectively, isolated from the ischemic (left) and nonischemic (right) kidneys.

### 2.3. Histological Score of Kidney (HSK)

The excised kidneys were fixed in phosphate-buffered 10% formalin, sectioned, and then stained with hematoxylin and eosin. Evaluation of HSK was performed in a blind manner by a pathologist. HSK was graded on a 4-point scale [2, 18]: 0 = normal histology; 1 = mild damage (less than onethird of nuclear loss (necrosis) per tubular cross section); 2 = moderate damage (greater than one-third and less than two-thirds of tubular cross section showing nuclear loss (necrosis)); 3 = severe damage (greater than two-thirds of tubular cross section showing nuclear loss (necrosis)). The total score per kidney section was calculated by addition of all 10 scores with a maximum possible injury score of 30.

### 2.4. Isolation of Kidney SP Cells

The protocol was based on a report by Challen et al. [6]. Briefly, anesthetized mice were perfused via the abdominal aorta with normal saline, and the minced kidneys were digested enzymatically in Hank's balanced salt solution (HBSS) (Invitrogen, Sweden) containing 1.2 U/mL Dispase II (Roche, Italy), 0.01% DNAse type I (Sigma, USA), and 7.5 mg/mL collagenase B (Roche) for 20 min at 37°C. The cell suspension was filtered through 40 *μ*m strainers (BD Falcon 2350; BD Pharmingen, USA) to remove debris and washed in HBSS containing 10 mmol/L HEPES (Sigma) and 2% fetal bovine serum (FBS) (Gibco, USA). Cells were resuspended in Dulbecco's modified Eagle's medium (DMEM) (HyClone, USA) containing 2% FBS, 10 mM HEPES, and 5 *μ*g/mL Hoechst 33342 (Sigma), with or without 50 *μ*M verapamil (Sigma), at 1 × 10^6^ cells/mL, incubated at 37°C for 90 min, and washed in cold phosphate-buffered saline (PBS) prior to cell surface antigen staining. Cell surface antigen staining was performed at 4°C for 20 min using monoclonal rat anti-mouse antibodies reactive to CD45-FITC, c-Kit-FITC, CD31-PE, and isotype controls (BD Pharmingen). Propidium iodide (PI, 2 *μ*g/mL) (BD Pharmingen) was added prior to fluorescence-activated cell sorting (FACS) analysis to exclude dead cells.

### 2.5. Cell Culture

Cells were plated in DMEM containing 10% FBS, 5 *μ*g/mL insulin, 5 *μ*g/mL transferrin, 5 pM thyronine, 50 nM hydrocortisone, 50 nM selenium, and 50 nM prostaglandin (all Sigma) at 37°C in a 95% air/5% CO_2_ incubator for 48 h. The conditions that might be observed in ischemia were simulated by hypoxia and by serum deprivation (SD) of the culture medium. For hypoxia exposure, cells were maintained under hypoxic conditions in an airtight chamber (Billups-Rothenberg, USA) containing a gas mixture composed of 95% N_2_ and 5% CO_2_ at 37°C for 6 h. For SD experiments, cells were washed with serum-free DMEM and placed in serum-free medium under normoxic or hypoxic conditions at 37°C for 6 h.

### 2.6. Apoptosis Assay

Apoptosis assay was performed by using an Annexin V = FITC apoptosis detection kit (BD Pharmingen) according to the manufacturer's instructions. In brief, cells were rinsed with ice-cold PBS, resuspended in binding buffer, incubated with Annexin V-FITC and PI in the dark for 15 min, and then immediately analyzed by using a FACScan flow cytometer.

### 2.7. Cell Cycle Assay

Cells were suspended in 1× PBS at a concentration of 1 × 10^6^ cells/mL and then ice-cold 70% ethanol was dropwise added into cell solution. After incubating overnight at 4°C, the cells were resuspended with paraformaldehyde fixation solution and fixed on the surface of ice for 1 h. Cells were then collected by centrifugation and resuspended in PI staining solution containing 0.05% Triton X-100, 100 *μ*g/mL RNase A, and 50 *μ*g/mL PI. After incubating for 30 min at 37°C, cells were immediately analyzed by using the FACScan flow cytometer.

### 2.8. FACS Analysis

Cell analysis and sorting were performed on a dual-laser FACS Vantage (BD Biosciences, USA). Hoechst 33342 was excited at 355 nm UV light, and fluorescence emission was collected with a 675 nm long-pass filter (Hoechst red) and a 450/20 band-pass filter (Hoechst blue), and a 610 nm dichroic mirror short-pass was used to separate the emission wavelengths. PE, FITC, and PI fluorescence was detected by using a 488 nm argon laser, and live cell gate was defined as the cells being excluded from the cells positive for PI.

### 2.9. Enzyme Linked Immunosorbent Assay (ELISA)

The production of vascular endothelial growth factor (VEGF), insulin-like growth factor 1 (IGF-1), hepatocyte growth factor (HGF), and stromal cell-derived factor-1 (SDF-1*α*) in the supernatants of SP and non-SP was determined by ELISA using a commercially available ELISA kit (R&D Systems, USA) according to the manufacturer's recommendation. About 1 × 10^6^ cell suspension was cultivated in serum-free or serum-containing DMEM medium for 6 h under the conditions of normoxia or hypoxia. Protein was quantified by BCA protein assay reagent (Pierce, USA). Optical density was measured at 450 nm with wavelength correction at 570 nm. All samples and standards were measured twice.

### 2.10. Statistical Analysis

Data are presented as mean ± SD. Differences between data means were checked by analysis of variance (ANOVA) or Student's *t*-test using the SPSS statistical software package (SPSS, Inc., Chicago, IL, USA). A *P* value of less than 0.05 was considered statistically significant.

## 3. Results

### 3.1. Depletion of Kidney SP Cells after Acute Renal IRI

**Figure 1:**
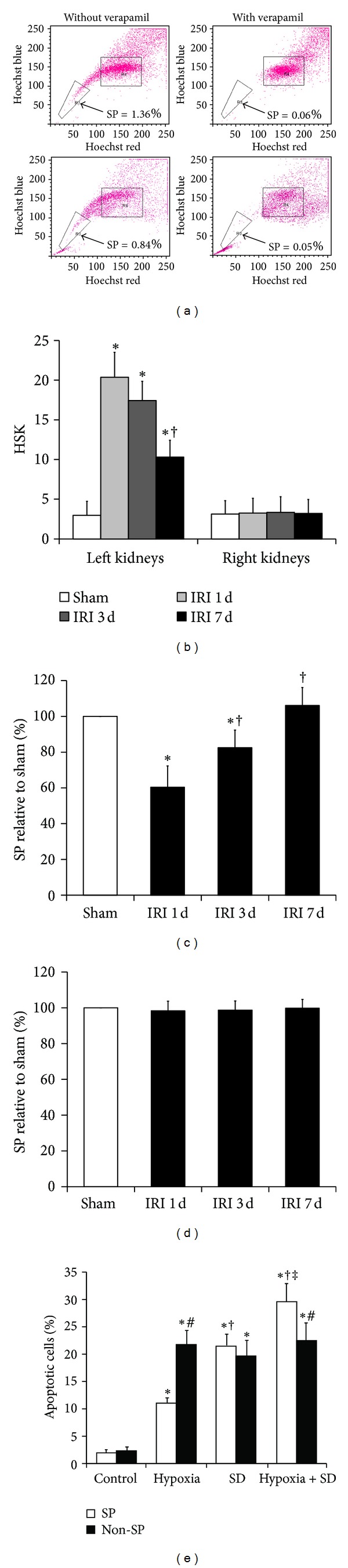
Depletion of SP cells in the ischemic kidney after IRI. (a) Fluorescence-activated cell sorting isolation of SP and non-SP cells in the left kidney from normal animals and IRI animals. Hoechst low SP cells are identified by the R3 area and demonstrate an a decrease in kidney SP cells within 1 day after IRI. (b) The histological score of kidney (HSK) in kidneys from sham-operated and IRI-mice was calculated. *P < 0.05 versus sham; ^†^ *P* < 0.05 versus IRI 1 d. ((c) and (d)) The SP cells in left (c) and right (d) kidneys from sham-operated (white bars) and IRI (black bars) animals. *P < 0.05 versus sham; ^†^ *P* < 0.05 versus IRI 1 d. (e) *In vitro* apoptosis analysis of kidney SP and non-SP cells in basal conditions and after simulated ischemia. Cultured kidney SP and non-SP cells were subjected to hypoxia and SD alone or in combination for 6 h. Bar graph described from the FACS-based Annexin V/propidium iodide apoptosis assay. The cells without both hypoxia and SD stimulation were used as controls. *P < 0.05 versus control; ^†^ *P* < 0.05 versus hypoxia; ^‡^ *P* < 0.05 versus SD; ^#^ *P* < 0.05 versus non-SP.

Whole kidney cells were isolated from C57BL/6 mice and stained with Hoechst 33342 dye (Figure 1(a)). FACS analysis of renal isolates from normal animals revealed that the Hoechst-extruding, verapamil-sensitive kidney SP cell fraction showed, on average, 1.36% of the total viable cell population (1.13% to 1.59%). Importantly, the kidney SP pools were significantly depleted by more than 40% within 1 day after IRI. To determine the response of endogenous kidney SP to ischemic kidney injury, adult animals received sham operation and unilateral renal IRI. Immunohistochemistry staining showed that left (ischemic) kidneys from mice at 1 d after IRI had significantly higher histological score of kidney (HSK) than sham-operated kidneys, and the increased HSK was decreased to 50.6 ± 9.1% at 7 d after IRI. In contrast, the HSK in the right (nonischemic) kidneys was not changed during all the observation times. All the above results indicated that the model for unilateral renal IRI had been successfully established (Figure 1(b)). In nonoperated and in sham-operated animals, regional kidney SP levels were similar in the left kidney (data not shown). Interestingly, after unilateral renal IRI, the ischemic (left) kidney, but not nonischemic (right) kidney, showed an acute depletion of SP cells within one day (Figures 1(c) and 1(d)). Importantly, left kidney SP cells were progressively restored to baseline levels within 7 days after IRI. For right kidney, a percentage of the kidney SP cells had no change over time.

### 3.2. *In Vitro* Apoptosis of Kidney SP Cells with Hypoxia and SD Alone or in Combination

Since hypoxia and SD are both components of ischemia-reperfusion *in vivo*, we further investigated the impact of the two components on kidney SP and non-SP cell apoptosis *in vitro*. To achieve this goal, kidney SP and non-SP cells were incubated in four kinds of culture conditions for 6 h as follows: DMEM medium supplemented with or without 10% FBS under normoxia or hypoxia (Figure 1(e)). Under hypoxia with FBS condition, it caused more apoptotic (Annexin V^+^) cells (in both SP and non-SP groups) than under normoxia with FBS condition; however, hypoxia caused fewer apoptotic cells in SP group than in non-SP group. Meanwhile, under normoxia without FBS, it caused more apoptotic cells (similarly in both SP and non-SP groups) than under normoxia with FBS. Finally, the hypoxia without FBS condition generated the most apoptotic cells among four kinds of culture conditions. In one word, SD alone increased the level of apoptosis both in SP and non-SP cells, and hypoxia further aggravated the SD-induced cell apoptosis.

### 3.3. The Paracrine Actions of Kidney SP Cells after IRI

**Figure 2:**
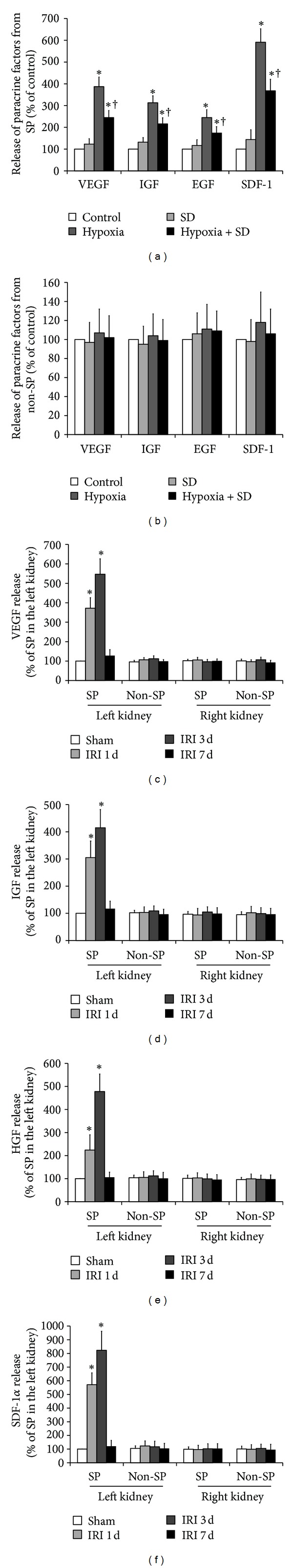
Hypoxia induces the increase in SP cell paracrine actions *in vitro* and *in vivo*. ((a) and (b)) Effect of hypoxia and SD alone or a combination of both on SP and non-SP cell paracrine actions *in vitro*. Cultured kidney SP (a) and non-SP (b) cells were subjected to hypoxia and SD alone or in combination for 6 h, and then the secretion levels of VEGF, IGF-1, HGF, and SDF-1*α* were quantified by ELISA. The cells without both hypoxia and SD stimulation were used as controls. *P < 0.05 versus control; ^†^ *P* < 0.05 versus hypoxia. ((c)–(f)) ELISA was performed to detect the secretion levels of VEGF, IGF-1, HGF, and SDF-1*α* in the SP and non-SP cells separated from the ischemic (left) and nonischemic (right) kidneys of unilateral renal IRI mice. *P < 0.05 versus sham.

We first determined the effects of hypoxia on the secretion of chemokines and mitogenic factors in kidney SP cells. The result showed that hypoxia for 6 h significantly increased the release of VEGF, IGF-1, HGF, and SDF-1*α* when compared with normoxia in the serum-containing or serum-free media (Figure 2(a)). However, SD markedly reduced the hypoxia-induced increase in the production of these factors (Figure 2(a)). Furthermore, the secretion levels of these factors in non-SP cells did not change by hypoxia and/or SD (Figure 2(b)). Next, we wanted to determine whether hypoxia *in vivo* induced SP cell paracrine actions. In the left kidney, the secretion of growth factors in the SP but not in non-SP cells appeared to be increased within the first day of IRI, peaked on the third day, and rapidly downregulated on the seventh day after injury to the level observed in sham-operated kidneys (Figures 2(c)–2(f)). In the right kidney, however, the secretion levels of these factors in both SP and non-SP cells had no change over time.

### 3.4. The Proliferation of Kidney SP Cells after IRI

**Figure 3:**
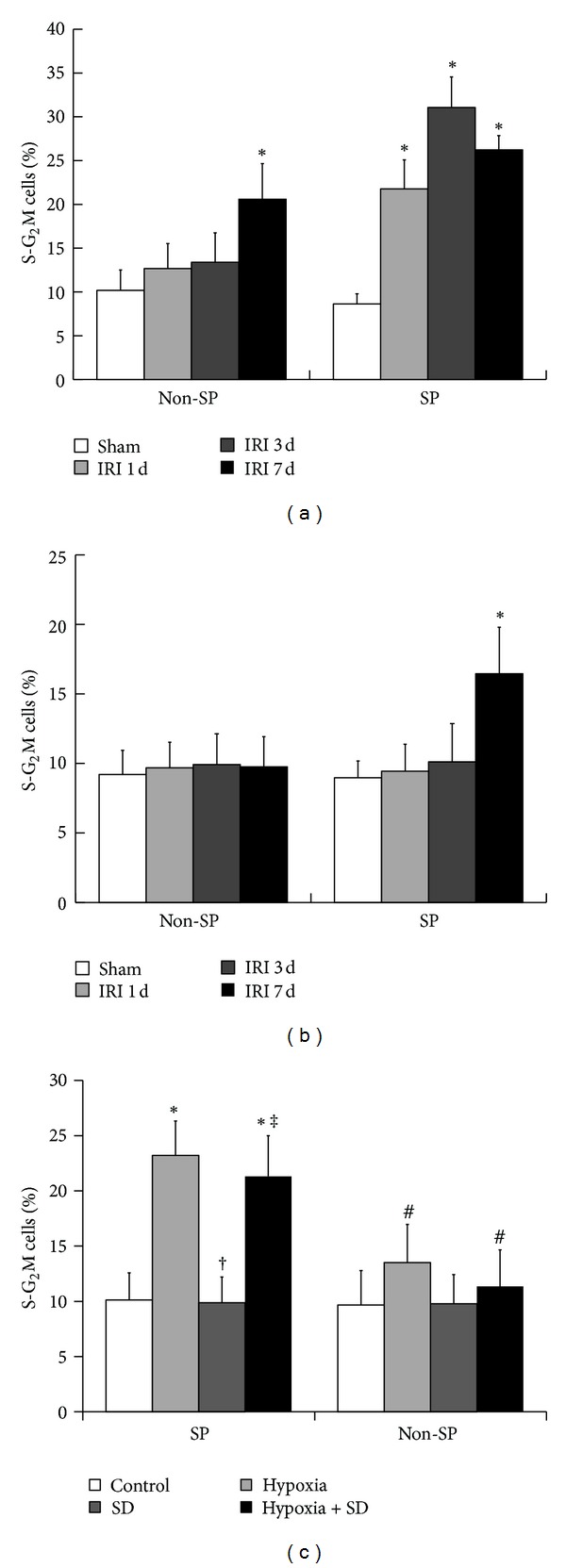
Proliferation of SP cells in the ischemic kidney after IRI. ((a) and (b)) Proliferation of the SP and non-SP cells in the ischemic (a) and nonischemic (b) kidney after IRI. Fractions of S-G_2_M kidney SP and non-SP in sham-operated and IRI animals were obtained by propidium iodide staining. *P < 0.05 versus sham. (c) Effect of hypoxia and SD alone or a combination of both on cell proliferation *in vitro*. The cells without both hypoxia and SD stimulation were used as controls. *P < 0.05 versus control; ^†^ *P* < 0.05 versus hypoxia; ^‡^ *P* < 0.05 versus SD; ^#^ *P* < 0.05 versus SP.

The cellular mechanisms being responsible for reconstituting kidney SP following IRI remain unknown. We, therefore, quantified kidney SP and non-SP in S-G_2_M phase from sham-operated and IRI animals, using the cell cycling marker, the DNA binding dye, PI. After IRI, the SP cells in left kidney underwent self-proliferation and reentered the cell cycle on the first day, as marked by a 2.5-fold increase in PI, and remained cycling on the seventh day after IRI (Figure 3(a)). The proliferation of non-SP in left kidney was delayed, with increased PI staining on the seventh day after injury (Figure 3(a)). In the right kidney, the SP proliferation was also delayed and non-SP proliferation had no change over time (Figure 3(b)). In addition, the role of either hypoxia or SD *in vitro* in the proliferation of kidney SP and non-SP cells was also evaluated. The result showed that hypoxia but not SD induced the increase in proliferation *in vitro* in kidney SP cells. However, neither hypoxia nor SD had effect on the proliferation of the kidney non-SP cells (Figure 3(c)).

### 3.5. Phenotype of Kidney SP Cells after IRI

**Figure 4:**
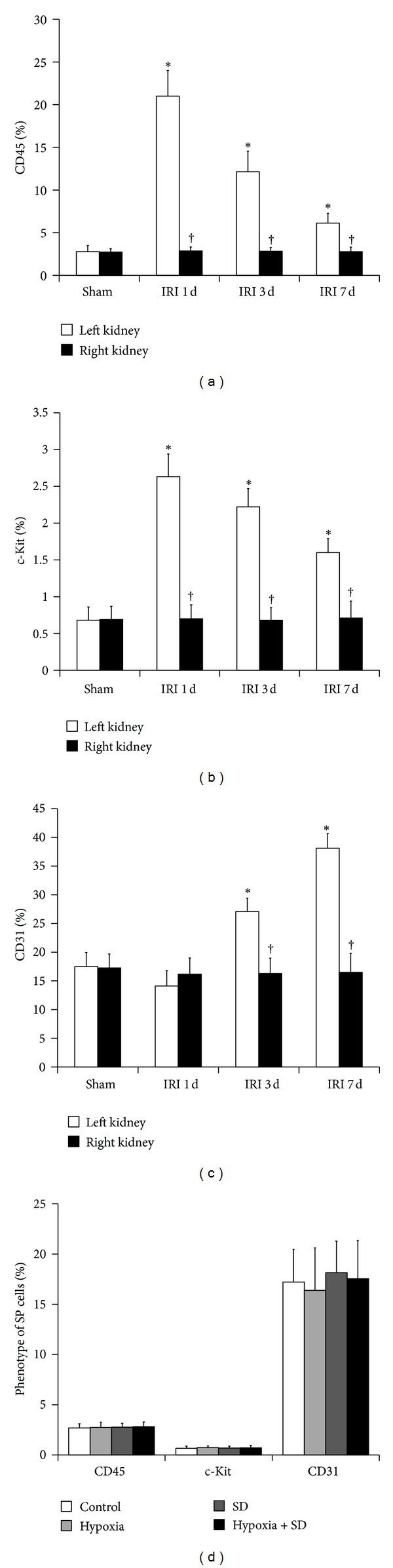
Phenotype of SP cells in the ischemic kidney after IRI. ((a)–(c)) The expression of CD45 (a), c-Kit (b), and CD31 (c) in left (white bars) and right (black bars) kidney SP from sham-operated and IRI animals. *P < 0.05 versus sham. ^†^ *P* < 0.05 versus left kidney. (d) Effect of hypoxia and SD alone or a combination of both on the expression of CD45, c-Kit, and CD31 *in vitro*. The cells without both hypoxia and SD stimulation were used as controls.

Characterization by flow cytometry confirmed that the SP cells from normal kidney expressed CD31 (17.5% ± 2.7%) and lacked hematopoietic stem cell markers such as CD45 (2.8% ± 0.7%) and c-Kit (0.7% ± 0.4%). In the kidney SP cells isolated from mice on the first day after IRI, the percentage of cells expressing CD45 and c-Kit was observed to increase in the left kidney compared with the right kidney (Figures 4(a) and 4(b)), suggesting the hematopoietic origin and bone marrow phenotype of these cells. With time following renal injury, the number of CD45 and c-Kit positive cells significantly decreased. Furthermore, CD31 expression was low among left kidney SP on the first day after IRI but increased significantly within 7 days (Figure 4(c)). In the right kidneys after IRI, the percentage of SP cells expressing CD45, c-Kit, and CD31 had no change over time (Figures 4(a)–4(c)). In addition, as shown in Figure 4(d), neither hypoxia *in vitro* nor SD *in vitro* had effect on the expression of CD45, c-Kit, and CD31 in the kidney SP cells. In short, these results suggested the possibility about the BMC homing to ischemic kidney and about their immunophenotypic conversion from BMCs to kidney SP cells.

## 4. Discussion

SP cells have been found in various types of adult tissues including kidney and are presumed to be a stem cell-rich population [4–13]. Protective effects of kidney SP cells have been demonstrated by the findings that infusion of exogenous kidney SP cells can improve renal function in different models of renal injury [6, 7, 9, 12]. However, experiments based on the administration of exogenous SP do not necessarily reflect the physiological role of endogenous SP, and the response of the endogenous SP to renal injury remains incompletely understood.

We first identified the existence of SP cells in adult C57BL/6 mice kidneys and an acute depletion of kidney SP cells on the first day following renal IRI. Next, we wanted to determine whether hypoxia and SD, which were both components of ischemia *in vivo*, could induce the increase in SP apoptosis. As expected, either hypoxia or SD alone increased the level of SP cell apoptosis and a combination of both further aggravated it. Importantly, the hypoxia-induced increase in apoptosis was much less in SP cells than that in non-SP cells, suggesting that SD was the stronger one in kidney SP cells between the two stimuli on cell apoptosis. Because the SP cells were more survivable than non-SP cells under hypoxia conditions, we hypothesized that molecules or proteins secreted by SP cells could inhibit hypoxia-induced cell apoptosis. As expected, our result showed that, at least in SP cells, hypoxia *in vitro* but not SD *in vitro* induced the enhanced secretion of VEGF, IGF-1, HGF, and SDF-1*α*. Furthermore, the increased secretion of growth factors was discovered in the SP but not non-SP cells in the ischemic kidney after acute renal IRI. These data suggest the potential role of SP cell paracrine actions in repair after acute renal IRI.

We also evaluated serial changes of kidney SP number during acute renal IRI and, for the first time, discovered that the initial depleted SP cells in the ischemic kidney were progressively restored to baseline levels within 7 days after IRI. Although the clear evidence has showed that renal recovery from ischemic injury requires regeneration of damaged tubular epithelium [14], the relative relevance of regeneration of kidney SP cells in physiopathological conditions still needs to be identified further. In the present study, we confirmed that the proliferation of SP cells was earlier than that of non-SP cells in the ischemic kidney and was delayed in the nonischemic kidney, suggesting that the proliferation of resident kidney SP cells might partially participate in the renewal of kidney SP cells following renal IRI.

Furthermore, immunophenotypic analysis showed that the SP cells obtained from normal kidneys contained very low levels of cells that expressed CD45 and c-Kit, which were predominant in bone marrow SP cells, suggesting that the kidney SP cells, under physiologic conditions, were a resident nonhematopoietic cell population. Marumo et al. [13] confirmed that kidney SP fraction was reduced by 38% in tubulointerstitial injury caused by unilateral ureteral obstruction, and the number of cells expressing CD45 in kidney SP cells was conversely increased in obstructed kidneys. Although the possibility that BMCs might functionally contribute to the renal regeneration after inducing renal ischemia was still a matter of debate, mobilization of BMCs to injured kidney was clear [19–23]. To determine the possible contribution of SP fraction within BMCs to the injured kidney, the expression of CD45 and c-Kit on SP cells from kidneys was serially investigated. Our results showed that the expression of CD45 and c-Kit was significantly increased in the SP fraction in kidneys from IRI animals compared with sham-operated animals on the first day after surgery, suggesting that the SP cells of BMCs, which were positive for CD45 and c-Kit, might be mobilized and home to ischemic kidney and partly contribute, at least, to maintain and reconstitute the kidney SP pools under renal IRI. In addition, with time following renal injury, the percentage of cells expressing CD45 and c-Kit decreased significantly and the percentage of cells expressing CD31 (vascular endothelial cells marker) increased significantly within 7 days, suggesting that the SP cells in ischemic kidney, which probably originated from resident renal cells or BMCs, could undergo tissue-specific immunophenotypic conversion to participate in restoration of ischemic renal tissue.

In conclusion, these studies demonstrate that the initial depleted kidney SP cells are progressively restored to baseline levels within 7 days after IRI. The reconstitution of this cell population may come from the proliferation of existing SP cells and homing of bone marrow-derived SP cells to ischemic kidney. The kidney SP cells can be used as a therapeutic target which can modulate tissue regeneration in AKI by promoting their activation or preventing their depletion. Further investigation into the function and biological signals of the reconstituted SP cell population following injury is of clinical importance to promote renal regeneration.
